# Comparison between multiparametric MRI with and without post - contrast sequences for clinically significant prostate cancer detection

**DOI:** 10.1590/S1677-5538.IBJU.2018.0102

**Published:** 2018

**Authors:** Thais Caldara Mussi, Tatiana Martins, George Caldas Dantas, Rodrigo Gobbo Garcia, Renee Zon Filippi, Gustavo Caserta Lemos, Ronaldo Hueb Baroni

**Affiliations:** 1 Departamento de Radiologia e Diagnóstico por Imagem, Hospital Israelita Albert Einstein, SP, Brasil; 2 Ecoar Medicina Diagnóstica, Lourdes, Belo Horizonte, MG, Brasil; 3 Departamento de Intervenção Guiada por Imagens, Hospital Israelita Albert Einstein, SP, Brasil; 4 Departamento de Patologia, Hospital Israelita Albert Einstein, SP, Brasil; 5 Departamento de Urologia, Hospital Israelita Albert Einstein, SP, Brasil

**Keywords:** Magnetic Resonance Imaging, Prostatic Neoplasms, Men

## Abstract

**Background:**

Dynamic-contrast enhanced (DCE) sequence is used to increase detection of small lesions, based on increased vascularization. However, literature is controversy about the real incremental value of DCE in detection of clinically significant (CS) prostate cancer (PCa), since absence of enhancement does not exclude cancer, and enhancement alone is not definitive for tumor. Purpose: To test the hypothesis that DCE images do not increase CS PCa detection on MRI prior to biopsy, comparing exams without and with contrast sequences. Material and Materials and Methods: All men who come to our institution to perform MRI on a 3T scanner without a prior diagnosis of CS PCa were invited to participate in this study. Reference standard was transrectal prostate US with systematic biopsy and MRI/US fusion biopsy of suspicious areas. Radiologists read the MRI images prospectively and independently (first only sequences without contrast, and subsequently the entire exam) and graded them on 5-points scale of cancer suspicion.

**Results:**

102 patients were included. Overall detection on biopsy showed CS cancer in 43 patients (42.2%), clinically non-significant cancer in 11 (10.8%) and negative results in 48 patients (47%). Positivities for CS PCa ranged from 8.9% to 9.8% for low suspicion and 75.0% to 88.9% for very high suspicion. There was no statistical difference regarding detection of CS PCa (no statistical difference was found when compared accuracies, sensitivities, specificities, PPV and NPV in both types of exams). Inter-reader agreement was 0.59.

**Conclusion:**

Exams with and without contrast-enhanced sequences were similar for detection of CS PCa on MRI.

## INTRODUCTION

Prostate cancer (PCa) is a major global health problem, as the most common cancer in men, aside from skin cancer, and the second - leading cause of cancer death in the United States (1). Approximately 30% of men older than 50 years of age have pathologic evidence of PCa; however, only 3% will die from their disease (2, 3). The diagnosis of PCa increased in the mid 1980's when prostate - specific antigen (PSA) became a screening biomarker. However, PSA screening is a cause of over diagnosis and consequent overtreatment of patients with indolent disease (2). Therefore, the recommendation to use PSA for PCa screening remains controversial (4).

Efforts have been made to better define the clinical behavior of prostate tumors, which can range from indolent and clinically insignificant (CI) tumors to aggressive and metastatic cancer (5, 6).

Benefits of multiparametric magnetic resonance imaging (mpMRI) in patients with clinical suspicion of PCa are already established (7). MpMRI has the ability to improve detection of clinically significant (CS) PCa and decrease the detection of CI tumors prior to biopsy (7-9). Some studies already demonstrated that mpMRI is the best predictor for CS PCa detection (10, 11). Additionally, mpMRI used along with PSA has been shown to increase negative predictive values to rule out PCa, making it an excellent test to avoid unnecessary biopsies in biopsy - naïve patients and men with prior negative biopsies (12-14).

A routine mpMRI should include T1 - weighted (T1W), T2 - weighted (T2W), diffusion - weighted image (DWI), and dynamic contrast - enhanced (DCE) sequences, as recommended by major international guidelines (15). T1W images are used to detect hemorrhages within the prostate and seminal vesicles. T2W images are mostly used to evaluate prostatic anatomy, detect morphological abnormalities, and evaluate extraprostatic extension and seminal vesicle invasion in cases of advanced tumors. DWI is helpful to differentiate CS PCa from benign lesions and predict cancer aggressiveness. It should be used in conjunction with the other sequences. Finally, DCE is used to increase detection of small lesions (13), based on increased vascularization of these lesions. However, the real incremental value of DCE in detection of CS PCa is controversial, since absence of enhancement does not exclude cancer, and enhancement alone is not definitive for tumor (16).

Regardless of its advantages and increased usefulness, mpMRI is expensive and time consuming Gadolinium introduces risk of allergic reaction, potential development of nephrogenic systemic fibrosis and deposition in brain tissue (11, 17-19). However, the clinical effects of deposition of this agent contrast in the brain are not know until nowadays.

The objective of our study is to test the hypothesis that contrast - enhanced images do not increase the detection of CS PCa on mpMRI prior to biopsy, comparing exams with and without contrast in the same patient population.

## MATERIALS AND METHODS

### Study design

From June 2015 until February 2016, all male patients who came to our institution to perform prostatic mpMRI without a prior diagnosis of CS PCa were invited to participate in this prospective, institutional review board approved study (CAAE number 40942915.7.0000.0071). All male patients included in this study signed informed consent.

Exclusion criteria were: prostate biopsy not performed or performed in another institution, incomplete mpMRI protocol, biopsy performed more than six months after mpMRI, and an exam that was not evaluated by the two radiologists of this study.

A total of 447 patients signed the informed consent to enter the study over a nine month period, and 345 were excluded for the following reasons: prostate biopsy not performed or performed in another institution (n = 339), incomplete mpMRI protocol (n = 2), biopsy performed more than six months after mpMRI (n = 1), and exams not read not by the two study radiologists (n = 3).

### Imaging

All patients underwent mpMRI on a 3 - Tesla scanner: Magnetom Prisma (Siemens Medical Solutions, Erlangen, Germany) or Discovery MR 750W (GE Healthcare, Little Chalfont, United Kingdom) with a phased array coil and without an endorectal coil. A routine protocol including triplanar T2W imaging, DWI (b - values = 50, 400, 800 and 1500) and DCE sequences were performed covering the prostate and seminal vesicles. Fifteen post - contrast sequences were acquired with a temporal resolution of 13 seconds each. Extracellular gadolinium - based contrast media (Magnevist, Bayer, Leverkusen, Germany) was injected at a dose of 0.2 cc / Kg and a rate of 2 cc / sec.

### Biopsy protocol

As reference standard, transrectal prostate ultrasound (US) systematic biopsy (14 - cores, 12 from peripheral zone and two from transition zone) and mpMRI / US fusion with additional samples of suspicious areas was adopted. US - guided biopsies were performed using either an Aplio 500 with Smart Fusion (Toshiba Medical System Corporation, Minato, Tokyo, Japan) or a LOGIC E9 with imaging fusion software (GE Healthcare, Little Chalfont, United Kingdom). One out seven radiologists with experience in prostate biopsy with imaging fusion mpMRI / US (minimum of 3 year of experience) performed the prostate biopsy, aware of mpMRI findings.

TOne out four of the pathologists from the hospital performed the histopathologic analysis, with at least 15 years of experience in uropathology. Histological findings were classified for each prostatic region as negative, positive CI tumor (Gleason 3 + 3), or positive CS tumor (Gleason ≥ 3 + 4) (20).

### Data analysis

Two fellowship trained radiologists (with 6 and 15 years of experience in prostate mpMRI) read images prospectively and independently (blinded to each other): first they filled in a form classifying the prostate mpMRI in suspicion levels for PCa reading only sequences without contrast. Subsequently, they filled in another form reclassifying the suspicion levels for PCa reading the entire exam including the post - contrast enhancement sequences. Both radiologists were aware of the patient's clinical data. Analysis was performed into eight prostatic regions (apex, mid and base of peripheral zone; transition zone, right and left), and graded on 5 - point scale of cancer suspicion (1: CS PCa is very unlikely; 2: CS is unlikely; 3: presence of CS PCa is equivocal; 4: CS PCa is likely; and 5: CS PCa is very likely). A final consensus analysis was performed to make the final report, which was used to guide the suspicious areas on biopsies. The imaging-pathologic correlation was performed by one of the authors after all the MRI readings were finished (18).

### Statistical methods

We performed a histogram analysis to verify the distribution. Because numeric variables were not normally distributed, they were described with median and interquartile range (IQR).

To verify the association between mpMRI categories (1-5) and biopsy results we used generalized estimating equations (21), with permutable correlation structures, using the software R 3.1.3 (R Core Team, 2015). Sensitivity, specificity, positive predictive value (PPV), negative predictive value (NPV) and accuracy for both readers were calculated using biopsy as reference standard. The level for statistical significance was set at 5%.

Inter - reader agreement was calculated using Cohen's Kappa coefficient of agreement within ordinal weights, and it was defined as: excellent (k ≥ 0.81), good (k = 0.61 – 0.80), moderate (k = 0.41 – 0.60), fair (k = 0.21 – 0.40), and poor (k ≤ 0.20).

## RESULTS

The final cohort was comprised of 102 patients with a median age of 62.1 years old (range 35.1 – 82.1). Median time between mpMRI and biopsy was 15 days (IQR 14; 16); median PSA level was 4.36 ng / mL (IQR 3.19; 5.83); median number of fragments in the prostate biopsy was 19 (IQR 17; 21); and median number of fragments for each suspicious lesion was 4 (IQR 3; 5). Twenty - five patients (24%) were submitted to prior biopsy, and of those, 19 (76%) had negative results. The remaining 6 patients (24%) were on active surveillance for a CI tumor (up to two fragments of Gleason 3 + 3 on previous biopsy). Previous prostate biopsies were performed with a median time of 21 months (range 2 – 180) prior the mpMRI and those patients had no post - biopsy hemorrhage in the prostate gland during exam analysis.

Overall biopsy results showed CS cancer in 43 (42.2%), CI cancer in 11 (10.8%), and negative result for cancer in 48 (47%) patients. Of the 25 patients who had prior biopsies with negative results or CI tumors, 10 (40%) had new diagnoses of CS tumors and one (4%) maintained CI tumor diagnosis.

**Figure 1:**
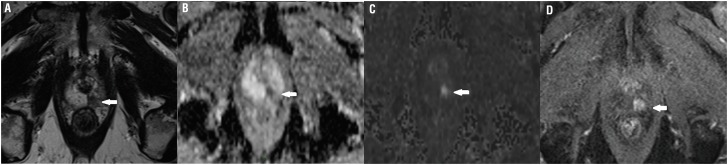
Seventy three years old man with PSA level of 3.4 ng/mL and normal DRE. MpMRI shows a 7 mm nodule in the left apical peripheral zone (T2-weighted imaging on A), with marked restricted diffusion seen on ADC map (B) and on b-value of 1500 (C). The lesion was categorized as very high suspicion for CS PCa (category 5) for both readers in both reading sessions despite the small size and DCE sequence. Early enhancement of the lesion is seen on DCE (D). Biopsy confirmed Gleason 4+4.

Each radiologist evaluated a total of 816 prostatic regions in each phase of the study (eight prostatic regions in 102 patients). Table-1 provides the mpMRI readings on the eight prostatic regions that had no statistical difference regarding detection of CS PCa in exams with and without contrast for both readers. Positive CS PCa ranged from 8.9% to 9.8% for low suspicion (category 2) and 75.0% to 88.9% for very high suspicion (category 5) on mpMRI categories (Figure-1). The odds of having CS PCa on mpMRI was 2.75 (reader 1) and 2.4 (reader 2). In corroborating these findings, no statistical difference was found when we compared accuracy, sensitivity, specificity, PPV, and NPV in both sets of exams (Table-2). Accuracy was slightly better in exams without contrast for both readers, but without statistical significance.

**Table 1:** Positivity results regarding the suspicion level on mpMRI in a sextant pattern.

Radiologist	MRI category	Contrast		Biopsy								
			Global	Negative			Positive clinically nonsignificant			Positive clinically significant		
			N	n	p (95%CI)	p-value	n	p (95%CI)	p-value	n	p (95%CI)	p-value
1	1	With	10	10	100.0	–	0	0.0	–	0	0.0	–
		Without	10	10	100.0		0	0.0		0	0.0	
	2	With	504	435	86.3 (83.3-89.3)	0.969	21	4.2 (2.4-5.9)	0.868	48	9.5 (7.0-12.1)	0.876
		Without	530	457	86.2 (83.3-89.2)		21	4.0 (2.3-5.6)		52	9.8 (7.3-12.3)	
	3	With	247	202	81.8 (77.0-86.6)	0.813	9	3.6 (1.3-6.0)	0.877	36	14.6 (10.2-19.0)	0.731
		Without	230	190	82.6 (77.7-87.5)		9	3.9 (1.4-6.4)		31	13.5 (9.1-17.9)	
	4	With	36	20	55.6 (39.3-71.8)	0.187	2	5.6 (0.0-13.0)	0.984	14	38.9 (23.0-54.8)	0.185
		Without	28	11	38.9 (20.9-56.9)		2	5.7 (0.0-13.6)		15	55.8 (37.1-74.5)	
	5	With	19	3	15.8 (0.0-32.2)	0.679	0	0.0	–	16	84.2 (67.8-100.0)	0.679
		Without	18	2	11.1 (0.0-25.6)		0	0.0		16	88.9 (74.4-100.0)	
2	1	With	9	9	100.0	–	0	0,0	–	0	0.0	–
		Without	12	9	75.0		0	0,0		3	25.0	
	2	With	482	418	86.7 (83.7-89.8)	0.819	19	3.9 (2.2-5.7)	0.984	45	9.3 (6.7-11.9)	0.799
		Without	485	423	87.2 (84.2-90.2)		19	3.9 (2.2-5.6)		43	8.9 (6.3-11.4)	
	3	With	268	212	79.1 (74.2-84.0)	0.851	10	3.7 (1.5-6.0)	0.924	46	17.2 (12.6-21.7)	0.801
		Without	257	205	79.8 (74.9-84.7)		10	3.9 (1.5-6.3)		42	16.3 (11.8-20.9)	
	4	With	38	27	71.1 (56.6-85.5)	0.673	3	7.9 (0.0-16.5)	0.899	8	21.1 (8.1-34.0)	0.590
		Without	42	28	66.7 (52.4-80.9)		3	7.1 (0.0-14.9)		11	26.2 (12.9-39.5)	
	5	With	19	4	21.1 (2.7-39.4)	0.770	0	0.0	–	15	78.9 (60.6-97.3)	0.770
		Without	20	5	25.0 (6.0-44.0)		0	0.0		15	75.0 (56.0-94.0)	

**Mp =** multiparametric; **MRI =** magnetic resonance imaging; **N =** number of prostate regions; **95%CI =** 95% confidence intervals; P-value to compare exams without and with contrast.

**Table 2:** Diagnostic measurements.

Radiologist	Contrast	Accuracy	P-value	Sensitivity	P-value	Specificity	P-value	PPV	P-value	NPV	P-value
1	With	65.2 (61.9-68.5)	0.346	57.9 (48.8-67.0)	0.594	66.4 (62.9-69.9)	0.208	21.9 (17.2-26.5)	0.860	90.7 (88.1-93.2)	0.872
	Without	67.4 (64.2-70.6)		54.4 (45.2-63.5)		69.5 (66.1-72.9)		22.5 (17.5-27.4)		90.4 (87.9-92.9)	
2	With	63.1 (59.8-66.4)	0.837	60.5 (51.6-69.5)	0.892	63.5 (60.0-67.1)	0.781	21.2 (16.8-25.7)	0.979	90.8 (88.3-93.4)	0.961
	Without	63.6 (60.3-66.9)		59.6 (50.6-68.7)		64.2 (60.7-67.8)		21.3 (16.8-25.8)		90.7 (88.2-93.3)	

**PPV =** Positive Predictive Value; **NPV =** Negative Predictive Value.

The best sensitivity and specificity values were obtained when including category 3 (equivocal) as positive on mpMRI studies with and without contrast (Table-3).

**Table 3:** Diagnostic measures according to the Likert categories on mpMRI, for both radiologists, for exams read with and without the dynamic post-contrast sequences.

Exam	Reader	Category +	Sensitivity	Specificity
With contrast	1	2	100.0	1.4 (0.5-2.3)
		3	57.9 (48.8-67.0)	66.4 (62.9-69.9)
		4	26.3 (18.2-34.4)	96.4 (95.1-97.8)
		5	14.0 (7.7-20.4)	99.6 (99.1-100.0)
	2	2	100.0	1.3 (0.4-2.1)
		3	60.5 (51.6-69.5)	63.5 (60.0-67.1)
		4	20.2 (12.8-27.5)	95.2 (93.6-96.7)
		5	13.2 (7.0-19.4)	99.4 (98.9-100.0)
Without contrast	1	2	100.0	1.4 (0.5-2.3)
		3	54.4 (45.2-63.5)	69.5 (66.1-72.9)
		4	27.2 (19.0-35.4)	97.9 (96.8-98.9)
		5	14.0 (7.7-20.4)	99.7 (99.3-100.0)
	2	2	97.4 (94.4-100.0)	1.3 (0.4-2.1)
		3	59.6 (50.6-68.7)	64.2 (60.7-67.8)
		4	22.8 (15.1-30.5)	94.9 (93.2-96.5)
		5	13.2 (7.0-19.4)	99.3 (98.7-99.9)

Post - contrast sequences changed the overall mpMRI analysis in 11 cases for reader 1 (increasing the category in 10 cases) and in 7 cases for reader 2 (increasing the category in two cases). For reader 1, the post - contrast sequences correlated with biopsy results (positive enhancement in CS tumors or negative enhancement in negative results / CI tumors) in 5 cases (45%) and resulted in misclassification in 6 (55%). For reader 2, the sequences correlated with biopsy results in two cases (29%) and resulted in misclassification in five (71%). Post - contrast sequences identified four regions with CS tumors more than the exam without post - contrast sequences (4 / 114 = 3.5%) for reader 1 and one region more (1 / 114 = 0.9%) for reader 2. On the other hand, the change of the classification for mpMRI positive (categories 3 to 5) with post - contrast sequences had negative results on biopsy in 22 regions (22 / 670 = 3.3%) for reader 1 and in five regions (5 / 670 = 0.7%) for reader 2 (Table-1).

A total of 1632 prostatic regions were evaluated by each readers, and the inter - reader agreement was 0.59 (CI: 0.55 – 0.64), demonstrating good agreement. The inter - reader agreement in the per - patient analysis (a total of 102 patients in each exam phase for each reader) was 0.47 (CI: 0.31 – 0.64) on exams without post - contrast sequences and 0.54 (CI: 0.38 – 0.70) on exams with post - contrast sequences, demonstrating moderate agreement.

## DISCUSSION

Due to over - diagnosis and overtreatment of PCa in the PSA era, mpMRI became useful to detect and characterize prostatic lesions in patients with clinical suspicion for cancer prior to biopsy (7-10, 22). The use of MRI to detect CS PCa is already established by many studies performed with complete protocol of mpMRI, including contrast (23-25). Recent studies performed with a complete mpMRI protocol have demonstrated the benefits of MRI over some biomarkers for the detection of PCa (10) and to monitor candidates for active surveillance (26, 27).

As a non - invasive method used for prostatic tumor detection, ideally mpMRI should be as faster and cheaper as possible. It is known that contrast - enhanced mpMRI is more expensive, time - consuming, and increases the risk of potential allergic reactions, nephrogenic systemic fibrosis, and gadolinium brain tissue deposition (17-19).

In this prospective study we found similar detection rates for CS PCa in exams read with and without contrast - enhanced sequences with no statistical differences for the five levels of suspicion on mpMRI.

Two recent studies showed high accuracy of MRI for the detection of CS PCa, using a Likert scale with only T2W images and DWI (biparametric - MRI) and PSA levels (28, 29). These studies were retrospective, did not categorize the mpMRI suspicion level, and did not compare the results of biparametric - MRI with the gold standard of mpMRI (that includes post - contrast images). In our cohort we included all patients with clinical suspicion of PCa and all mpMRI exams regardless the suspicion level, which probably explains the higher specificity and NPV and lower sensitivity and PPV of our study when compared to their results.

Vargas et al., aiming to evaluate the recommendations in the PI - RADS version 2 and investigate the impact of pathologic tumor volume on PCa detectability on mpMRI, found limited added value of DCE to T2W and DWI sequences (30). Also, few studies showed similar performance for mpMRI with and without contrast media for PCa detection, using both Likert (31, 32) and PIRADS (33) scales. These findings corroborate ours that non - contrast mpMRI can improve PCa detection in the near future.

On the other hand, in a study that included only PI - RADS categories 3 and 4, Druskin et al. showed higher positivity for CS PCa in lesions category 3 with and without enhancement (upgraded to PI - RADS 4); however, both lower compared to PI - RADS 4 (32). This finding shows that a PI - RADS 3 lesion with positive enhancement (which is upgraded to PI - RADS 4) has lower risk of CS PCa than a PI - RADS 4 lesion, as showed in a prospective analysis performed by Mertan et al. (34).

We used a Likert scale to stratify the suspicion level on mpMRI, where the radiologist provided a score based on overall impression instead of a fixed criterion. The grade of diffusion restriction (low, moderate, and high) was the most important criteria to classify risk of CS PCa. When this study was designed PI - RADS version 2 had not been published (13) and PI - RADS version 1 was not in use at our institution. The Likert criteria was already shown to be more accurate when applied by readers with previous experience (35). Our study showed moderate to good rates of inter - reader agreement, similar values of those demonstrated using the PI - RADS classification (36-38).

Our study shows that the use of contrast in mpMRI does not increase the detection rate of CS PCa, and has similar accuracy, sensitivity, specificity, PPV, and NPV as compared to a non - contrast protocol. In this prospective study, we included all patients with no prior diagnosis of CS PCa. The diagnostic results yielded consistently high NPV to rule - out CS PCa (> 90%), which could help avoid unnecessary biopsies in patients with low suspicion on mpMRI (categories 1 and 2). Accuracy and specificity were slightly better for non - contrast exams for both readers, but without statistical significance.

This study had several limitations. First, since our institution is an open hospital, a high number of patients (345) did not perform biopsy at our institution and were excluded. Second, our population study included all patients without diagnostic of CS PCa (biopsy naïve, with negative previous biopsy and in active surveillance) and we did not perform a subgroup analysis. Also, we did not use the PIRADS classification; however, previous studies showed good performance of mpMRI using a Likert classification. We used biopsy as a reference standard instead of prostatectomy specimen, what could introduce an imaging - pathology correlation bias; however, many studies were published using this same methodology, with consistent results (39-41). We did not separate peripheral zone and transitional zone tumors. Our temporal resolution for DCE sequences was 13 seconds instead of 10 seconds recommended nowadays. Finally, the short time between the readings could introduce an interpretation bias, but such bias would favor the reading of images that included the contrast - enhanced series, which was performed at the end of the reading session.

In conclusion, our study shows similar performances of mpMRI with and without DCE for CS PCa detection. Further studies should be performed to confirm these results and confirm that a limited, faster, and cheaper mpMRI protocol can be used as standard technique.
